# Non-Technical Skills for Surgery in African Perioperative Curricula: A Scoping Review

**DOI:** 10.21203/rs.3.rs-11125425/v1

**Published:** 2026-10-08

**Authors:** Shaliz Barzani, Marie Merci Cyuzuzo, Wendy Williams, Egide Abahuje, Robert Riviello, Barnabas Tobi Alayande

**Affiliations:** Harvard University; University of Global Health Equity; Brigham and Women’s Hospital; University of Michigan; Harvard University; University of Global Health Equity

**Keywords:** Non-technical skills, Surgical training, Curricula, Surgical education, Surgery, Obstetrics and gynecology, Anesthesia, Midwifery, Nursing

## Abstract

**Introduction:**

Non-technical skills (NTS) such as situation-awareness, decision-making, communication, and leadership among surgical healthcare providers are vital to safe surgical care. Studies have associated poor NTS with errors in the operating room. The extent to which African training curricula address NTS is unclear. We mapped the presence and absence of NTS content in surgery, obstetrics and gynecology, anesthesia, and nursing/midwifery training curricula across Africa.

**Methods:**

Using the Arksey and O’Malley methodological framework, we conducted a scoping review on PubMed, AJOL, and Google Scholar databases, followed by a grey literature search to identify published perioperative training curricula from African institutions. Data on NTS were abstracted and were categorized as explicitly teaching, explicitly mentioning, implicitly mentioning, or making no mention of NTS.

**Results:**

From 55 screened documents, 31 specialty curricula from 18 institutions across 11 countries met inclusion criteria. West Africa was represented at 45.2%, followed by East Africa at 29.0%, and North and South Africa at 6.5% each. Curricula from the College of Surgeons of East, Central and Southern Africa, the College of Anesthesiologists of East, Central and Southern Africa, the East, Central and Southern African College of Obstetrics and Gynecology, and the WHO Africa Prototype Competency-Based Curriculum accounted for 12.9%. No single-country NTS curriculum was identified from Central Africa. NTS were mentioned implicitly (38.7%), explicitly (48.4%), both implicitly and explicitly (6.5%), or not at all (6.5%). Most curricula (93.5%) mentioned at least one NTS. Among NTS domains, leadership was most frequently mentioned (67.7%), followed by teamwork (61.3%), communication (45.2%), professionalism (35.5%), decision-making (32.3%), and problem-solving (29.0%). Critical thinking and situation awareness (6.5%) were the least represented domains.

**Conclusion:**

While several African perioperative curricula mention NTS, gaps exist in the incorporation of formalized NTS training in existing courses and assessments, especially in Central Africa. Targeted curriculum reform is needed to transition NTS from implicit expectations to explicitly taught and assessed competencies in African perioperative training. Structured coursework, such as the Non-Technical Skills for Surgeons in Variable Resource Contexts (NOTSS-VRC) may help fill these gaps.

## Introduction

Post-operative mortality is the third leading cause of death worldwide. Despite being young and healthy, African surgical patients are two times more likely than global counterparts to die after surgery, and about one in five African patients develop complications following planned surgeries.^1,2^ Causes of post-operative complications are multifactorial and include human performance and system-related factors. Human performance related factors include surgeons’ technical and non-technical skills. Several studies suggest that half of surgical adverse events stem from non-technical skills—the cognitive, social, and interpersonal skills that complement technical surgical expertise.^3,4,5^ In order to reduce perioperative adverse events in Africa, both technical and non-technical skills need to be developed and scaled.

Over the last few decades, efforts to improve access to surgical care in Africa and other resource-limited settings have increased significantly. There have been developments and expansions of surgical specialty training programs at the national (e.g., medical school-based residency training programs) and regional level (e.g., the College of Surgeons of East, Central, and Southern Africa and the West African College of Surgeons). Additional interventions to improve patient safety in African countries have consisted of developing educational interventions to enhance surgical teams’ non-technical skills performance.^6^

There have been a handful of these interventions, but one specifically adopted often is the Non-Technical Skills for Surgeons (NOTSS) taxonomy.^7^ NOTSS demonstrates the necessary content and accurately predicts the risk of adverse events in surgery. NOTSS-VRC is an adapted framework developed in Rwanda that addresses the peculiarities of a resource-variable context, accounting for factors such as unreliable electricity, water, imaging, blood products, staffing, and other resources.^6–9^ Other non-technical skill frameworks include Anaesthetists’ Non-Technical Skills (ANTS), Scrub Practitioners List of Non-Technical Skills (SPLINTS), Observational Teamwork Assessment for Surgery (OTAS), Oxford NOnTECHnical Skills (Oxford NOTECHS), Revised Non-Technical Skills scale (Revised NOTECHS), Nontechnical skills for anaesthetic trainees, Paediatric Cardiac Surgery Teamwork classification tool (PCST), Nurse Anaesthetists Nontechnical Skills – Denmark (NANTSdk), Objective Structured Assessment of Nontechnical Skills (OSANTS), and the WHO Behaviourally Anchored Rating Scale (WHOBARS), among others.^10^ Several NTS frameworks are being integrated into formal surgical training and continuing medical education globally.^11^

Despite the documented impact of non-technical skills on post-operative outcomes, surgical training curricula have traditionally only focused on teaching technical skills and medical knowledge. While several tools and taxonomies have been developed to assess and improve the non-technical skills of surgical care providers, it remains unclear to what extent these non-technical skills have been incorporated into African surgical training curricula. Understanding the non-technical skills content covered in African Medical education curricula is essential to ensure sustained efforts to improve perioperative patient safety. In this context, we wanted to ask, “What is the extent to which, and how, non-technical skills training is incorporated into surgical, obstetrics and gynecology, anesthesia, midwifery, and nursing curricula in Africa?” We aimed to systematically identify and characterize gaps in the incorporation of non-technical skills within perioperative (surgical, obstetrics and gynecology, anesthesia, and nursing) curricula across Africa. The population of interest was trainees and providers in postgraduate and undergraduate surgery, obstetrics and gynecology, anesthesia, and nursing/midwifery training programs based in Africa. The context was the presence, absence, and manner of incorporation of non-technical skills (NTS) — situation awareness, decision-making, communication, leadership, problem solving, critical thinking, professionalism, and teamwork — within training curricula. Context entailed formal perioperative training curricula (surgery, OBGYN, anesthesia, nursing/midwifery) published or currently in use by African institutions, regional colleges, or pan-continental bodies, at the undergraduate, medical, or postgraduate level, from 2015 onward.

## Methods

Using the Arksey and O’Malley framework, we conducted a scoping review on PubMed/MEDLINE, African Journals OnLine (AJOL), and Google Scholar databases followed by a grey literature search using Google to identify published perioperative training curricula from African institutions.^12^ An *a priori* unregistered protocol guided this scoping review (Additional file 1). The review is reported using the PRISMA-Scoping Review (PRISMA-ScR) extension checklist, and is available as (Additional file 2).

### Eligibility criteria

Curricula in any language were included in the review if they were designed for training in surgery and surgical subspecialties, obstetrics and gynecology, anesthesia, or nursing at the undergraduate, medical, or postgraduate training level. Any literature on NTS that was not a curriculum document was excluded (including simulation manuals, workshop guides, learning assessments, resident or course evaluations). Curricula designed and implemented outside of Africa were excluded, even if African trainees participated. As a proxy for reviewing only more recent curricula, all documents published before 2015 were excluded. Any non-extractable formats (broken links, inaccessible content) or unretrievable records following a 4-week period were excluded following unsuccessful attempts and consultation with a surgical education expert on the study team.

### Information sources

We searched PubMed/MEDLINE, AJOL, and Google Scholar. Grey literature search was conducted using Google Search. Expert recommendation by an African surgical education expert directed the researchers to the websites of pertinent regional and national surgical healthcare training colleges for further information.

### Search strategy

Database searches were conducted by two investigators (SB and MMC) between 20 July and 06 August 2026. Pubmed was searched using the terms ((“surgery curriculum” OR “surgical curriculum” OR “obstetrics and gynecology curriculum” OR “anesthesia curriculum” OR “nursing curriculum” OR “midwifery curriculum”) AND (Africa)). AJOL was also searched using “surgery curriculum” OR “surgical curriculum” OR “obstetrics and gynecology curriculum” OR “anesthesia curriculum” OR “nursing curriculum” OR “midwifery curriculum” without “Africa” since AJOĽs collection is Africa-focused by default. Google Scholar searches were limited to the 2015–2026 date range, consistent with our eligibility criteria. Because Google Scholar ranked results by relevance rather than allowing database-specific filtering, and returned a very high total record count (n = 6,478), screening was limited to the first 200 relevance-ranked records by title. This cutoff was applied after an initial review of the first 50 records confirmed no eligible curricula, following established guidance on the practical limitations of Google Scholar for systematic and grey-literature searching. The guidance similarly recommends restricting screening to an initial subset of relevance-ranked results given the sharp decline in eligible-study yield beyond that point.^13^

For the grey literature search, Google’s search engine was used with a similar search string, but substituting “[country name]” for “Africa,” with one search per country and a focus on the first page of the search returned. A parallel search was made in French, Spanish, Portuguese, and Arabic. Google yielded 55 records. Expert recommendations were also used to identify relevant regional and national specialty colleges whose published curricula were subsequently reviewed directly.

### Selection of sources of evidence

Search results were imported into an Excel sheet for screening and tracking. Duplicate articles were removed manually. All records underwent stepwise title and abstract screening by two independent reviewers (SB, MMC), with disagreements resolved through discussion or consultation with a third reviewer (BA). The same process was applied to grey literature and content from Google. Full-text review was done by SB, with support from MMC. Full-text articles were retrieved through Harvard University’s library services.

### Data charting process and data items

Once the list of eligible sources was finalized, all curriculum documents were reviewed and categorized into an Excel sheet independently by SB and confirmed by MMC. Information recorded included country, whether a curriculum document was published, surgical healthcare specialty, information source (website, organization, database, or accreditation document), year published or of last update, teaching format (structured course, didactic, simulation, or online).

Each curriculum document was analyzed for references to NTS domains of situation-awareness, decision-making, communication, leadership, problem solving, critical thinking, professionalism, and teamwork. The incorporation, or lack of incorporation, of these domains were categorized as the following: implicit mention (indirect mention of an NTS, e.g. “can work well with others”), explicit mention including learning objectives (e.g. “expect the trainee to exhibit professionalism” or “trainees are required to have skills in decision-making”), explicit teaching (e.g. courses on communication skills), explicit assessment (e.g. an assessment based on a trainee’s performance in decision-making), or no mention at all. Critical appraisal of individual sources of evidence was not relevant to this scoping review.

### Synthesis of results

Results were synthesized and summarized using descriptive statistics of count and percentage and compared by specialty and region. Multinational curricula were not added to individual country counts, but rather presented as connected to African Union regions.

## Results

We identified a total of 6,556 records from databases and 55 records from websites, organizations, and expert recommendations. From the 274 documents screened, 31 specialty curricula from 18 institutions across 11 countries met the inclusion criteria and were identified from websites, organizations and expert recommendations (Fig. 1), (Additional file 3)^[14–44]^. Data used to obtain results was solely from the grey literature search as the database search yielded no curriculum documents that were used.

### Characteristics of included curricula

Of the 31 included curricula, four were from multinational/regional bodies. These four curricula (12.9%) were from the College of Surgeons of East, Central and Southern Africa (COSECSA), the College of Anesthesiologists of East, Central and Southern Africa (CANECSA), the East, Central and Southern African College of Obstetrics and Gynecology (ECSACOG), and the WHO Africa Prototype Competency-Based Curriculum. No single-country NTS curriculum was identified from Central Africa, and only two countries from North Africa (Egypt, Tunisia) had available curricula that met inclusion criteria. These regional bodies are organized by specialty, with COSECSA focusing on surgery, CANECSA focusing on anesthesia, ECSACOG focusing on OBGYN, WACS focusing on anesthesia and surgical oncology, and the WHO African Region focusing on surgery.

West Africa was the most represented region in all curricula at 45.2% (Ghana (8), Nigeria (3), Liberia (1), and WACS (2)). This was followed by East Africa at 29.0% (Uganda (2), Kenya (2), Zambia (4), and South Sudan (1), and North Africa (Egypt, Tunisia) and South Africa (South Africa, Botswana) at 6.5% each (Fig. 2). One curriculum falls under the WHO African Region, making up 3.2% of the total identified curricula.

### Specialty distribution of included curricula

Among the 31 included curricula, surgery (including surgical subspecialties) was the most represented specialty, and the least represented was nursing/midwifery at 6.5% (n = 2) (Fig. 3). The predominance of surgery curricula was driven by the Ghana College of Physicians and Surgeons (GCPS) subspecialty fellowship curricula (cardiothoracic, neurosurgery, pediatric surgery, plastic/reconstructive surgery, urology, vascular surgery, and colorectal surgery, in addition to general surgery).

### Level of training represented by included curricula

Of the 31 included curricula, the majority were postgraduate residency-level programs (67.7%, n = 21), followed by fellowship-level programs (29.0%, n = 9) (Fig. 4). One curriculum (3.2%) did not explicitly state its level of training.

### Non-technical skills domains included in African surgical curricula

Among NTS domains, leadership was most frequently mentioned (67.7%, n = 21), followed by teamwork (61.3%, n = 19), and communication (45.2%, n = 14) (Fig. 5). Situation awareness (6.5%, n = 2) was least mentioned.

NTS were either mentioned implicitly (38.7%), explicitly (48.4%), both implicitly and explicitly (6.5%), or not mentioned at all (6.5%) (Fig. 6). Most curricula (93.5%) mentioned at least one NTS implicitly or explicitly, however, fewer than half structurally incorporated NTS teaching via a course in the curricula. Only 2/31 (6.4%) curricula combined explicit mention as learning objectives with explicit assessment, and none combined explicit integration into teaching with assessment.

The teaching format and assessment of NTS differed widely amongst identified curricula. Of the eight NTS domains that were taught in 22% of the courses, 90% were taught within a defined course format. Delivery of NTS content was through online or virtual teaching in 6% of curricula that taught NTS.

Assessment methods in the curricula included multiple choice questions, OSCE (Objective Structured Clinical Examination), oral exams, and logbooks or portfolios created by students. Of the 31 curricula, multiple choice questions were the most commonly used format (29.0%, n = 9), followed by oral examinations (22.6%, n = 7), logbooks or portfolios (19.4%, n = 6), and objective structured clinical examinations (OSCE) (6.5%, n = 2). Fourteen curricula (45.2%) used at least one of these four formats; seven (22.6%) used another assessment format not captured by this list (e.g., questionnaires, practicum-based evaluation, multi-source feedback, or a generic assessment form), and ten (32.3%) did not specify an assessment format. One must note that these figures reflect general curriculum assessment infrastructure rather than NTS-specific assessment, which was identified in only 2 of 31 curricula (see Fig. 6)

## Discussion

The scoping review identified 31 curricula across 18 training institutions in 11 African countries, plus four multinational or pan-continental bodies that address NTS within surgical, obstetrics and gynecology, anesthesia, and perioperative nursing and midwifery training. Our findings suggest that while NTS competencies are broadly acknowledged across African surgical training programs, they remain inconsistently defined, unevenly distributed geographically, and rarely taught or assessed with the structure and specificity that frameworks such as NOTSS recommend.^6,45^

The geographic distribution of NTS within curricula was uneven. West Africa accounted for the largest share of curricula with NTS (45.2%), followed by East Africa (29.0%), while South and North Africa were each represented at only 6.5%. Central Africa had no single-country curriculum in the included set; its only representation came through regional bodies (COSECSA, CANECSA, ECSACOG) whose catchment spans multiple regions. This distribution may reflect differences in the operations of postgraduate medical education, publication practices, or the availability of grey literature online, rather than true differences in NTS teaching activity. By identifying gaps in surgical curricula, institutions can strengthen their education, and policies can be put into place to better guide institutions, universities, training organizations, and policy makers to strengthen surgical education and outcomes across the continent.

The distribution of NTS domains across the curricula varied. Leadership (68%) and teamwork (61%) were the most frequently cited NTS domains, while situation-awareness (6%) and critical thinking (16%) were rarely addressed. This pattern may reflect the ease with which leadership and teamwork map onto, or are evaluated with, existing competency frameworks (e.g., professionalism milestones, multidisciplinary collaboration), compared to situation-awareness and decision-making — the “cognitive” NTS domains — which have been shown to carry greater inter-rater variability even within validated assessment instruments such as NOTSS, and which require more deliberate curricular design and simulation-based teaching to institute and evaluate.^45^ Given that situation-awareness failures are a well-documented contributor to intraoperative adverse events, this gap may represent a meaningful blind spot in current training approaches across the continent.^46^

The extent to which African training curricula address NTS also varies. Nearly half of the curricula mentioned NTS competencies explicitly including as learning objectives but had no clear evidence of inclusion in structured teaching. Additionally, more than a third (38.7%) referenced NTS only implicitly, through generic language such as “[expects that a resident] works effectively in a team,” without using NTS terminology at all. This suggests that NTS is often treated as an assumed or hopeful byproduct of clinical training rather than a distinct, teachable, and assessable skill set. Non-technical skills must not be a second thought in training, but emphasized alongside technical surgical skills.^9^ Only 6% (2/31) included an NTS-specific assessment tool, calling for future focus on not only teaching but also evaluating surgical trainees on their use of non-technical skills.^47^ In addition, that no undergraduate curriculum documents were found suggests that publicly available, indexed information on NTS incorporation in African perioperative training is concentrated at the postgraduate level. Undergraduate curricula — where foundational NTS habits may first be introduced — remain largely undocumented or inaccessible in the published and grey literature.

### Implications

These findings support the development of a standardized, regionally adapted NTS curriculum and assessment framework for African surgical and perioperative training.^47^ The curriculum should translate learning objectives into actual course teaching and assessment. Curricula should focus on underrepresented domains (situation-awareness, critical thinking) and underrepresented regions (Central and North Africa). Existing tools such as NOTSS-VRC could be adapted rather than built from scratch, providing adaptation accounts for resource constraints and team structures specific to African surgical settings. Different methods used to assess non-technical skills should be used to evaluate trainee performance.^47^ Regional colleges should focus on teaching and assessing non-technical skills in a structured manner, and include NTS assessments and training in their curricula.

#### Limitations.

This review relied on publicly available grey literature and indexed sources; curricula that exist but are not published or are not discoverable through the search strategy used may be underrepresented. Institutions with more robust online publication practices (e.g., Ghana College of Physicians and Surgeons) are consequently overrepresented relative to their true share of African surgical training capacity. The review also could not assess teaching quality or trainee outcomes.Considering that no undergraduate curriculum documents were found, our search could have unintentionally targeted postgraduate education documents.

## Conclusion

This scoping review of curriculum documents on surgical, obstetrics and gynecology, anesthesia, and nursing/midwifery across Africa has revealed significant gaps in the implementation of non-technical skills across Sub-Saharan Africa. There is a need for educational institutions to not only include NTS in their learning objectives, but also to explicitly teach them as a structured course. Notably, the greatest deficit of NTS training is demonstrated in documents from Central, Northern, and Southern Africa. Focusing on the implementation of NTS would be beneficial to surgical trainees and the healthcare system in these regions. Underrepresented NTS domains were especially decision making, problem solving, critical thinking, and situation awareness. This implies the need to prioritize the implementation of NTS into African surgical curricula documents.

## Supplementary Material

Supplementary Files

This is a list of supplementary files associated with this preprint. Click to download.

- AdditionalFile1.docx
- AdditionalFile2.docx
- AdditionalFile3.xlsx

## Figures and Tables

**Figure 1:**
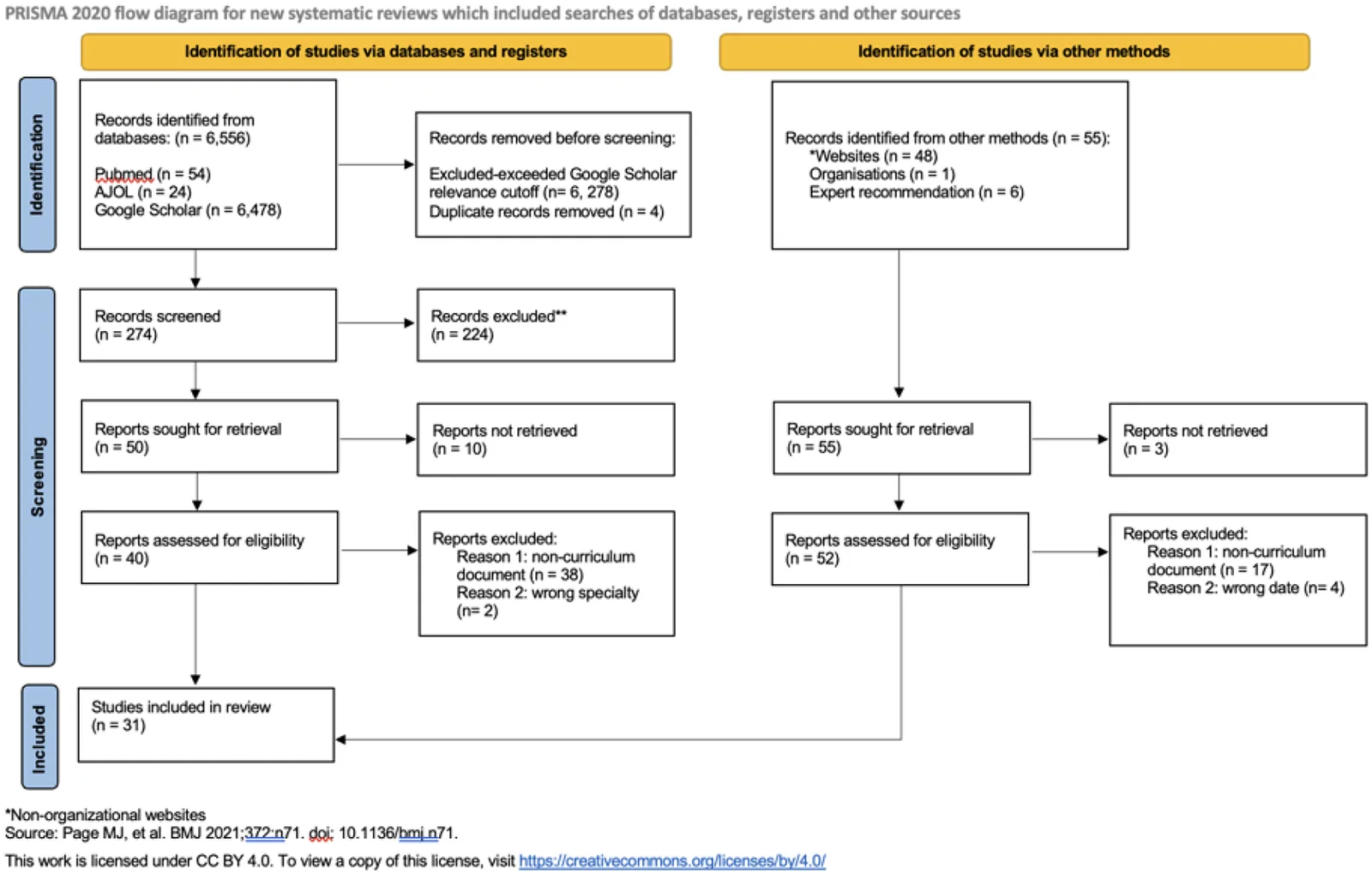
PRISMA flow diagram of Non-Technical Skills for Surgery in African Perioperative Curricula

**Figure 2:**
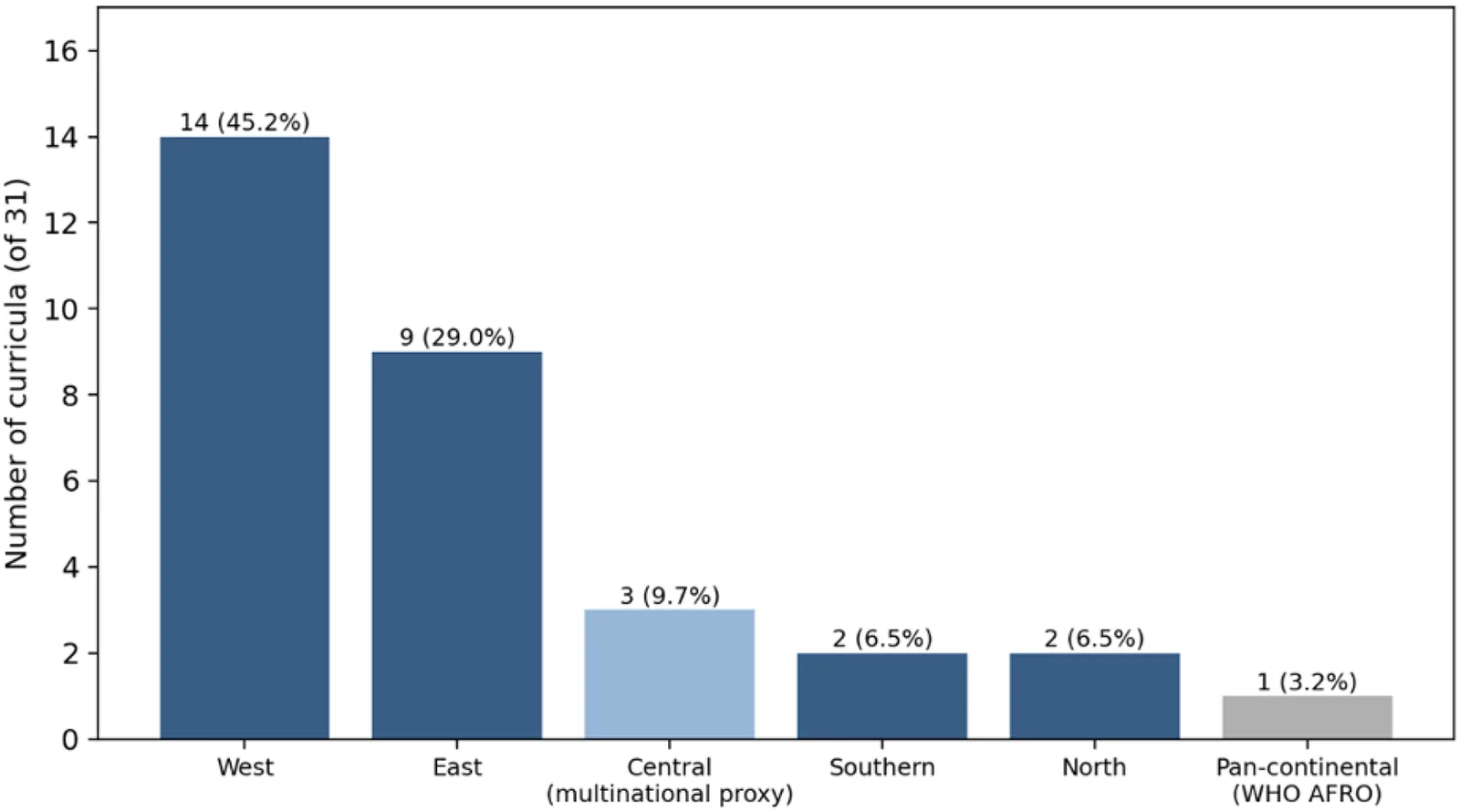
Distribution of identified curricula by African Union region.

**Figure 3:**
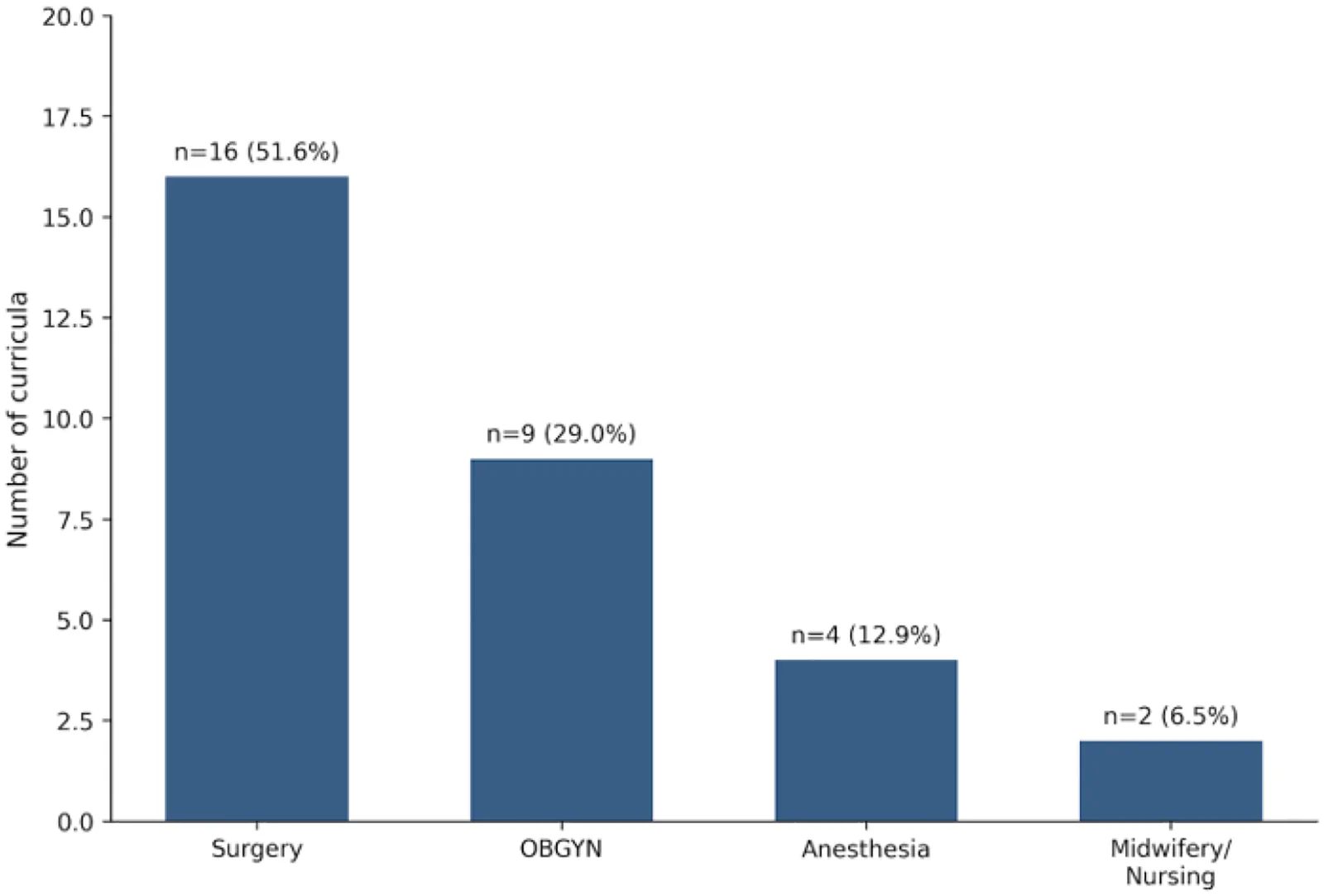
Distribution of curricula by surgical healthcare specialty.

**Figure 4:**
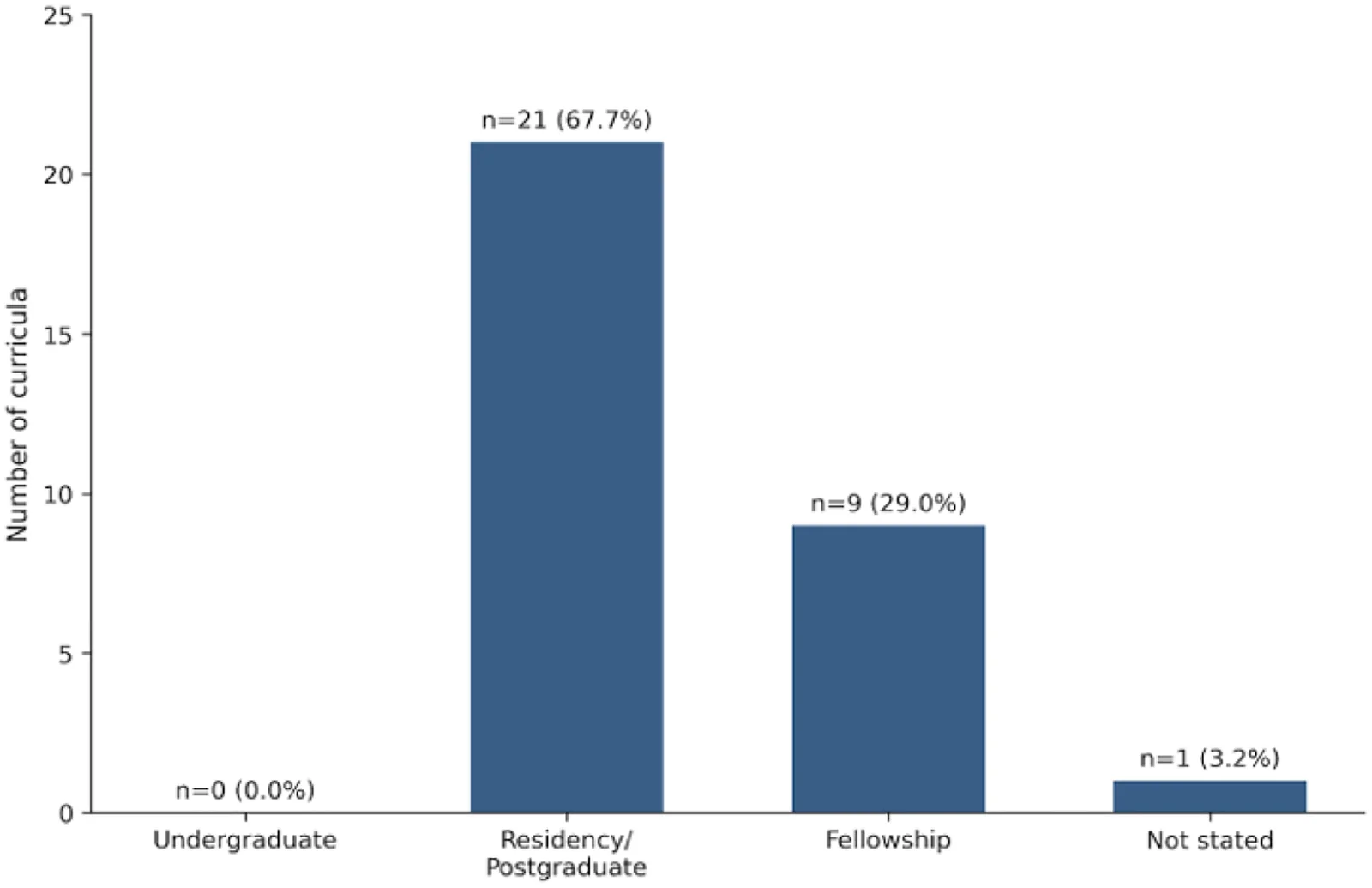
Distribution of surgical curricula by training level.

**Figure 5:**
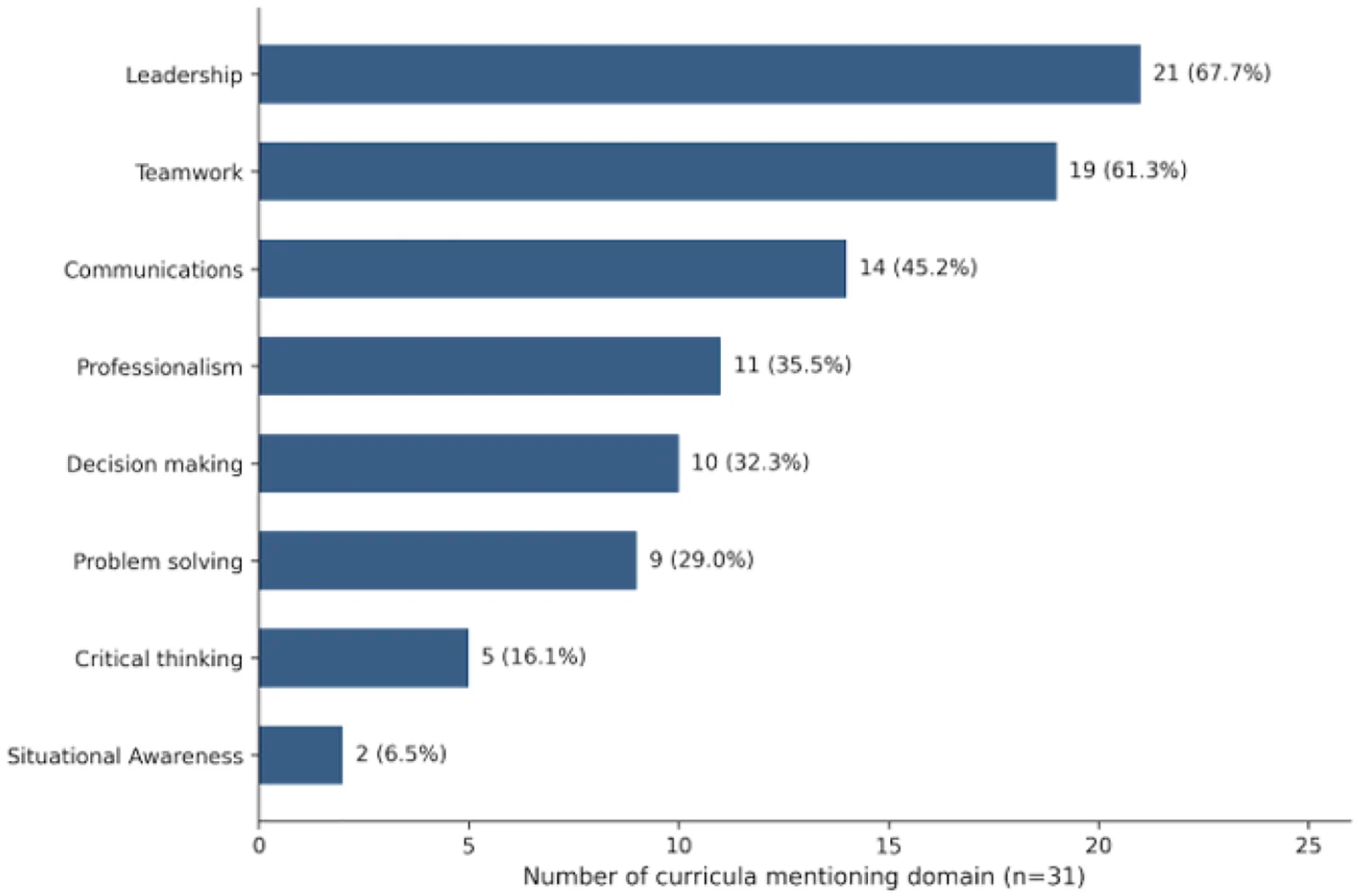
Frequency of non-technical skill domains across African Surgical Healthcare curricula.

**Figure 6:**
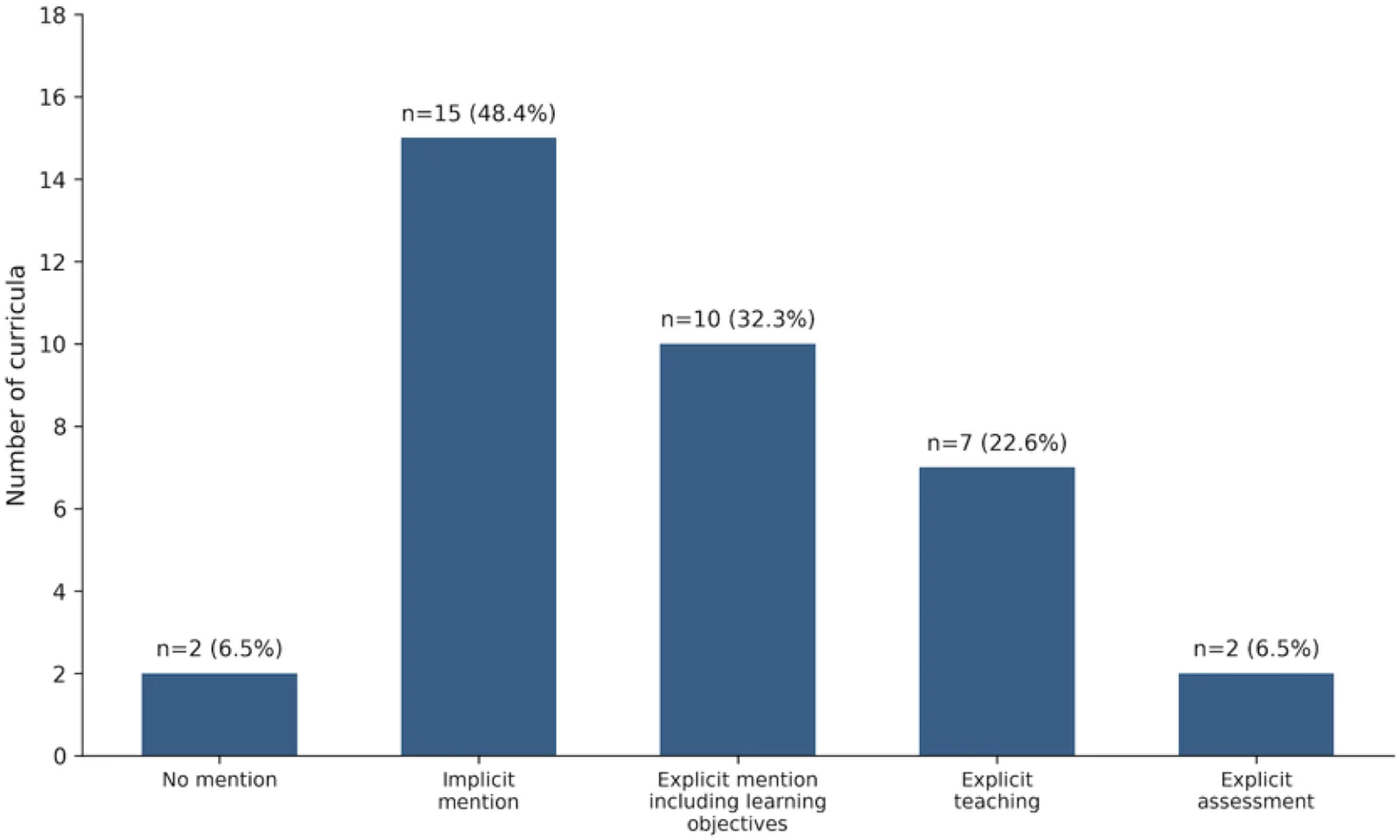
Distribution of how non-technical skills are represented across included African curricula (n=31)^a^ ^a^Categories in Figure 6 could incorporate NTS in more than one way at once (e.g., mentioning it explicitly while also assessing it, or mentioning it both implicitly and explicitly), so percentages sum to more than 100%.

## Data Availability

The data supporting the findings of this study are available within the article and its supplementary information.

## Abbreviations

NTS: Non-Technical skills
LCoGS: Lancet Commission on Global Surgery
NOTSS: Non-Technical Skills for Surgeons
ANTS: Anaesthetists’ Non-Technical Skills
SPLINTS: Scrub Practitioners List of Non-Technical Skills
OATS: Observational Teamwork Assessment for Surgery
Oxford NOTECHS: Oxford NOnTECHnical Skills
Revised NOTECHS: Revised Non-Technical Skills scale
PCST: Paediatric cardiac surgery teamwork classification tool
NANTSdk: Nurse Anaesthetists Nontechnical Skills – Denmark
OSANTS: Objective Structured Assessment of Nontechnical Skills
WHOBARS: WHO Behaviourally anchored rating scale
NOTSS-VRC: Non-Technical Skills for Surgery in Variable Resource Contexts
COSECSA: College of Surgeons of East, Central and Southern Africa
CANECSA: College of Anesthesiologists of East, Central and Southern Africa
ECSACOG: East, Central and Southern African College of Obstetrics and Gynecology
WHO: World Health Organization
OSCE: Objective Structured Clinical Examination

## References

[1] Biccard BM, Madiba TE, Kluyts HL, Munlemvo DM, Madzimbamuto F D, Basenero A, Perioperative patient outcomes in the African Surgical Outcomes Study: a 7-day prospective observational cohort study. The Lancet. 2018 Apr;391(10130):1589–98. 10.1016/S0140-6736(18)30001-1 29306587

[2] Meara JG, Leather AJM, Hagander L, Alkire BC, Alonso N, Ameh EA, Global surgery 2030: Evidence and solutions for achieving health, welfare, and economic development. The Lancet [Internet]. 2015 Aug [cited 2026 Sept 14];386(9993):569–624. Available from: https://www.thelancet.com/journals/lancet/article/PIIS0140-6736(15)60160-X/fulltext 10.1016/S0140-6736(15)60160-X 25924834

[3] Gawande AA, Zinner MJ, Studdert DM, Brennan TA. Analysis of errors reported by surgeons at three teaching hospitals. Surgery. 2003 June;133(6):614–21. 10.1067/msy.2003.169 12796727

[4] Suliburk JW, Buck QM, Pirko CJ, Massarweh NN, Barshes NR, Singh H, Analysis of Human Performance Deficiencies Associated With Surgical Adverse Events. JAMA Netw Open [Internet]. 2019 July 3 [cited 2026 Sept 14];2(7):e198067. Available from: https://pubmed.ncbi.nlm.nih.gov/31365107/10.1001/jamanetworkopen.2019.8067 PMC6669897 31365107

[5] Flin R, Yule S, McKenzie L, Paterson-Brown S, Maran N. Attitudes to teamwork and safety in the operating theatre. The Surgeon [Internet]. 2006 June [cited 2026 Sept 15];4(3):145–51. Available from: https://www.sciencedirect.com/science/article/pii/S1479666X06800843 10.1016/s1479-666x(06)80084-3 16764199

[6] Lin Y, Scott J, Mutabazi Z, Smirk D, Yule S, Riviello R, Strong support for a context-specific curriculum on non-technical skills for surgeons (NOTSS). East and Central African Journal of Surgery [Internet]. 2017 Jan 23 [cited 2026 Sept 20];21(3):3. Available from: https://www.ecajs.org/article/119227-strong-support-for-a-context-specific-curriculum-on-non-technical-skills-for-surgeons-notss

[7] Yule S, Paterson-Brown S. Surgeons’ Non-technical Skills. Surgical Clinics of North America [Internet]. 2012 Feb 1 [cited 2026 July 23];92(1):37–50. Available from: https://www.sciencedirect.com/science/article/pii/S0039610911001526 10.1016/j.suc.2011.11.004 22269259

[8] Non-technical skills for surgeons (NOTSS) | RCSEd [Internet]. Rcsed.ac.uk. 2026 [cited 2026 Sept 14]. Available from: https://www.rcsed.ac.uk/career-hub/learning-resources/non-technical-skills-for-surgeons-notss

[9] Lin Y, Scott JW, Yi S, Taylor KK, Ntakiyiruta G, Ntirenganya F, Improving surgical safety and nontechnical skills in variable-resource contexts: A novel educational curriculum. Journal of Surgical Education [Internet]. 2018 July [cited 2026 Sept 14];75(4):1014–21. Available from: https://www.researchgate.net/publication/320582078_Improving_Surgical_Safety_and_Nontechnical_Skills_in_Variable-Resource_Contexts_A_Novel_Educational_Curriculum 10.1016/j.jsurg.2017.09.014 29074364

[10] Wahr JA, Prager RL, Abernathy JH, Martinez EA, Salas E, Seifert PC, Patient safety in the cardiac operating room: Human factors and teamwork. Circulation [Internet]. 2013 Sept 3 [cited 2026 Sept 20];128(10):1139–69. Available from: https://www.ahajournals.org/doi/full/10.1161/cir.0b013e3182a38efa 10.1161/CIR.0b013e3182a38efa 23918255

[11] McMullan R D, Urwin R, Sunderland N, Westbrook J. Observational tools that quantify nontechnical skills in the operating room: A systematic review. J Surg Res [Internet]. 2020 Mar [cited 2026 Sept 20];247:306–22. Available from: https://pubmed.ncbi.nlm.nih.gov/31706538/10.1016/j.jss.2019.10.012 31706538

[12] Arksey H, O’Malley L. Scoping studies: Towards a methodological framework. International Journal of Social Research Methodology [Internet]. 2005 [cited 2026 Sept 20];8(1):19–32. Available from: https://www.tandfonline.com/doi/abs/10.1080/1364557032000119616

[13] Haddaway NR, Collins AM, Coughlin D, Kirk S. The Role of Google Scholar in Evidence Reviews and its Applicability to Grey Literature Searching. Wray KB, editor. PLOS ONE [Internet]. 2015 [cited 2026 Sept 18];10(9):e0138237. Available from: https://pmc.ncbi.nlm.nih.gov/articles/PMC4574933/10.1371/journal.pone.0138237 PMC4574933 26379270

[14] Parker AS, Hill KA, Steffes BC, Mangaoang D, O’Flynn E, Bachheta N, Design of a novel online, modular, flipped-classroom surgical curriculum for east, central, and Southern Africa. Ann Surg Open [Internet]. 2022 Mar 2 [cited 2026 Sept 18];3(1):e141. Available from: https://pubmed.ncbi.nlm.nih.gov/37600110/10.1097/AS9.0000000000000141 PMC10431259 37600110

[15] Bachelor of Medicine and Bachelor of Surgery - Makerere University School of Medicine [Internet]. Makerere University School of Medicine. 2022 [cited 2026 Sept 18]. Available from: https://som.mak.ac.ug/academic-programs/undergraduate-programs/bachelor-of-medicine-and-bachelor-of-surgery/

[16] Master of Medicine-Obs &Gyn [Internet]. Scribd. 2026 [cited 2026 Sept 18]. Available from: https://www.scribd.com/document/939744524/Master-of-Medicine-obs-Gyn

[17] Master of Medicine in General Surgery (mmed.surg) | Department of Surgery [Internet]. Uonbi.ac.ke. 2026 [cited 2026 Sept 18]. Available from: https://surgery.uonbi.ac.ke/programs/master-medicine-general-surgery-mmedsurg

[18] Zambia Colleges of Medicine & Surgery. Specialty Training Programme (STP) Curriculum & Learning Guide for General Surgery (2017) [Internet]. Zambia Colleges of Medicine & Surgery; 2017 [cited 2026 July]. Available from: https://www.zacoms.com/docs/stp_general_surgery_curriculum.pdf

[19] Zambia Colleges of Medicine & Surgery. Specialty Training Programme (STP) Curriculum & Learning Guide for Obstetrics and Gynaecology (2017) [Internet]. Zambia Colleges of Medicine & Surgery; 2017 [cited 2026 Aug]. Available from: https://www.zacoms.com/docs/stp_obgy_curriculum.pdf

[20] Zambia Colleges of Medicine & Surgery. Specialty Training Programme (STP) Curriculum & Learning Guide for Anaesthesia (2017) [Internet]. Zambia Colleges of Medicine & Surgery; 2017 [cited 2026 Aug]. Available from: https://www.zacoms.com/docs/stp_anaes_curriculum.pdf

[21] Diploma in Midwifery Curriculum [Internet]. Scribd. Nursing and Midwifery Council of Zambia; 2022 [cited 2026 July]. Available from: https://www.scribd.com/document/982317673/Final-NMCZ-Diploma-in-Registered-Midwifery-Curriculum-1

[22] West African College of Surgeons (WACS) Faculty of Anesthesia. Curriculum for Postgraduate Training in Anesthesia [Internet]. West African College of Surgeons (WACS); [cited 2026 July]. Available from: https://www.wacscoac.org/downloads/ANAESTHESIA%20CURRICULUM%202014.pdf

[23] College of Anaesthesiologists for East, Central and Southern Africa (CANECSA). Curriculum for the Fellowship in Anaesthesiology and Critical Care Medicine [Internet]. College of Anaesthesiologists for East, Central and Southern Africa (CANECSA); 2017 [cited 2026 July]. Available from: https://canecsa.org/wp-content/uploads/2024/11/CANECSA-Curriculum-2021.pdf

[24] The East, Central and Southern Africa College of Obstetrics and Gynecology (ECSACOG). Membership in Obstetrics and Gynecology (MCOG-ECSA) Curriculum [Internet]. The East, Central and Southern Africa College of Obstetrics and Gynecology (ECSACOG); 2017 [cited 2026 July]. Available from: https://ecsacog.org/wp-content/uploads/2025/07/Membership-Training-Curriculum-fin.pdf

[25] The Colleges of Medicine of South Africa NPC. Regulations for Admission to the Fellowship of the College of Surgeons of South Africa FCS(SA) [Internet]. The Colleges of Medicine of South Africa NPC; 2022 [cited 2026 July 21]. Available from: https://cmsa.co.za/wp-content/uploads/2024/02/FCSSA_Regulations_-_Effective_FS_2023_19_2_2024.pdf

[26] Directorate of Training and Professional Development Ministry of Health Republic of South Sudan. Diploma Midwifery Curriculum [Internet]. Directorate of Training and Professional Development Ministry of Health Republic of South Sudan; 2016 [cited 2026 July 21]. Available from: https://static1.squarespace.com/static/605888ceb52e0f614e2c2b4c/t/606b471634e46c200e3af863/1617643327694/NATIONAL+CURRICULUM+FOR+

[27] Ministry of Health & Population High Committee of Health Specialties Egyptian Fellowship Board. General Surgery Training Manual and Curriculum [Internet]. Scribd. 2020 [cited 2026 Sept 18]. Available from: https://www.scribd.com/document/749910900/GS-Training-Manual-and-Curriculum-May-2020-MG-FINAL

[28] Faculté de Médecine de Tunis. Curriculum des études médicales certificat de gynecologie obstetrique intégration spécialité intitulés durée total [Internet]. 2023 [cited 2026 July 22]. Available from: https://fmt.rnu.tn/wp-content/uploads/2025/01/GYNECOLOGIE-OBSTETRIQUE-fusionne.pdf

[29] Rosen B, Itsura P, Tonui P, Covens A, van Lonkhuijzen L, Orang’o EO. Development of a comprehensive and sustainable gynecologic oncology training program in western Kenya, a low resource setting. Gynecol Oncol Rep [Internet]. 2017 July 20 [cited 2026 July 22];21:122–7. Available from: https://pubmed.ncbi.nlm.nih.gov/28861459/10.1016/j.gore.2017.06.014 PMC5558466 28861459

[30] Luckett R, Nassali M, Melese T, Moreri-Ntshabele B, Moloi T, Hofmeyr GJ, Development and launch of the first obstetrics and gynaecology master of medicine residency training programme in Botswana. BMC Med Educ [Internet]. 2021 Jan 6 [cited 2026 Aug 1];21(1):19. Available from: https://pubmed.ncbi.nlm.nih.gov/33407415/10.1186/s12909-020-02446-1 PMC7789389 33407415

[31] National Postgraduate Medical College of Nigeria, Faculty of Anesthesia. Residency Training Curriculum [Internet]. National Postgraduate Medical College of Nigeria, Faculty of Anesthesia; 2021 [cited 2026 July 26]. Available from: https://npmcn.edu.ng/downloads/ANAESTHESIAFACCURRICULUM-REVISED2021.pdf

[32] National Postgraduate Medical College of Nigeria. Competency Based Curriculum for Junior Residency Training [Internet]. National Postgraduate Medical College of Nigeria; 2020 [cited 2026 July 26]. Available from: https://npmcn.edu.ng/downloads/FMCOGJUNIORRESIDENCYCURRICULUMFORSENATE.pdf

[33] National Postgraduate Medical College of Nigeria, Faculty of Obstetrics and Gynecology. Competency Based Curriculum for Senior Residency Training [Internet]. National Postgraduate Medical College of Nigeria; 2020 [cited 2026 July 26]. Available from: https://npmcn.edu.ng/downloads/SENIORRESIDENCYTRAININGCURRICULUMFORSENATE.pdf

[34] Ghana College of Physicians and Surgeons. Faculty of Surgery Fellowship Curriculum (General Surgery) [Internet]. Ghana College of Physicians and Surgeons. 2020 [cited 2026 July 23]. Available from: https://gcps.edu.gh/surgery-and-subspecialties/

[35] Ghana College of Physicians and Surgeons. Faculty of Surgery Fellowship Curriculum (Cardiothoracic Surgery) [Internet]. Ghana College of Physicians and Surgeons. 2020 [cited 2026 July 23]. Available from: https://gcps.edu.gh/surgery-and-subspecialties/

[36] Ghana College of Physicians and Surgeons. Faculty of Surgery Fellowship Curriculum (Neurosurgery) [Internet]. Ghana College of Physicians and Surgeons. 2020 [cited 2026 July 23]. Available from: https://gcps.edu.gh/surgery-and-subspecialties/

[37] Ghana College of Physicians and Surgeons. Faculty of Surgery Fellowship Curriculum (Paediatric Surgery) [Internet]. Ghana College of Physicians and Surgeons. 2020 [cited 2026 July 23]. Available from: https://gcps.edu.gh/surgery-and-subspecialties/

[38] Ghana College of Physicians and Surgeons. Faculty of Surgery Fellowship Curriculum (Plastic & Reconstructive Surgery) [Internet]. Ghana College of Physicians and Surgeons. 2020 [cited 2026 July 23]. Available from: https://gcps.edu.gh/surgery-and-subspecialties/

[39] Ghana College of Physicians and Surgeons. Faculty of Surgery Fellowship Curriculum (Urology) [Internet]. Ghana College of Physicians and Surgeons. 2020 [cited 2026 July 23]. Available from: https://gcps.edu.gh/surgery-and-subspecialties/

[40] Ghana College of Physicians and Surgeons. Faculty of Surgery Fellowship Curriculum (Vascular Surgery) [Internet]. Ghana College of Physicians and Surgeons. 2020 [cited 2026 July 23]. Available from: https://gcps.edu.gh/surgery-and-subspecialties/

[41] Ghana College of Physicians and Surgeons. Faculty of Surgery Fellowship Curriculum (Colo & Rectal Surgery) [Internet]. Ghana College of Physicians and Surgeons. 2020 [cited 2026 July 23]. Available from: https://gcps.edu.gh/surgery-and-subspecialties/

[42] Beddoe AM, Reis M, Benson A, Rehwaldt L, Mullbah J, Johnson J, Community-Institutional Partnerships to Strengthen Maternal Health Care: Case study of the First Obstetrics and Gynecology Specialty Training Program in Liberia. Frontiers in Public Health [Internet]. 2022 Feb 7 [cited 2026 Sept 18];9. Available from: https://pmc.ncbi.nlm.nih.gov/articles/PMC8858838/10.3389/fpubh.2021.779035 PMC8858838 35198530

[43] Nanji S, Ezeome E, Alatise O, Merchant S, Dedey F, Jalink M, A novel curriculum for surgical oncology fellowship training in West Africa. Ann Surg Oncol [Internet]. 2025 Mar [cited 2026 Sept 18];32(3):1590–600. Available from: https://pubmed.ncbi.nlm.nih.gov/39546103/10.1245/s10434-024-16348-9 39546103

[44] World Health Organization Regional Office for Africa. Prototype competency-based curriculum for general surgery [Internet]. World Health Organization; 2025. Available from: https://iris.who.int/server/api/core/bitstreams/51febaea-73d6-4d1a-a034-344a224199c8/content

[45] Gittins A. The utility of non-technical skills for surgeons (NOTSS) as an assessment tool in surgical training. Cureus. 2024 Nov 28;16(11). 10.7759/cureus.74676 PMC11604176 39611072

[46] Graafland M, Schraagen JMC, Boermeester MA, Bemelman WA, Schijven MP. Training situational awareness to reduce surgical errors in the operating room. British Journal of Surgery [Internet]. 2014 Oct 9 [cited 2026 Sept 20];102(1):16–23. Available from: https://onlinelibrary.wiley.com/doi/full/10.1002/bjs.9643 10.1002/bjs.9643 25298183

[47] Ounounou E, Abdullatif A, Brunckhorst O, Shamim KM, Dasgupta P, Kamran A. Nontechnical skills in surgery: A systematic review of current training modalities. Journal of Surgical Education [Internet]. 2019 Jan 1 [cited 2026 Sept 22];76(1):14–24. Available from: https://www.sciencedirect.com/science/article/pii/S1931720417307201 10.1016/j.jsurg.2018.05.017 30122636

